# Influence of X Elements on the Tribological Properties and Surface Chemistry of MXene Atomic Layers

**DOI:** 10.1021/acs.nanolett.5c02270

**Published:** 2025-06-07

**Authors:** Hong Yeon Yoon, Hyunjoon Yoo, Manmatha Mahato, Jong Hun Kim, Sokhna Dieng, Chi Won Ahn, Yury Gogotsi, Il-Kwon Oh, Jeong Young Park

**Affiliations:** ‡ Department of Chemistry, Korea Advanced Institute of Science and Technology, Daejeon 34141, Republic of Korea; § National Creative Research Initiative for Functionally Antagonistic Nano-Engineering, Department of Mechanical Engineering, Korea Advanced Institute of Science and Technology, Daejeon 34141, Republic of Korea; ⊥ Department of Physics, 26718 Inha University, Incheon 22212, Republic of Korea; ∥ Department of Materials Science & Engineering and A. J. Drexel Nanomaterials Institute, 6527 Drexel University, Philadelphia, Pennsylvania 19104, United States; ¶ National Nanofab Center (NNFC), Korea Advanced Institute of Science and Technology, 291 Daehak-ro, Yuseong-gu, Daejeon 34141, Republic of Korea

**Keywords:** 2D material, atomic force microscopy, MXene, friction, adhesion, X-ray photoelectron spectroscopy

## Abstract

MXenes, a class of two-dimensional (2D) transition-metal carbides, nitrides, and carbonitrides, exhibit promising tribological properties at the nanoscale. However, the influence of X elements on the surface chemistry of MXene atomic layers remains underexplored. Here, we investigate how nitrogen in the Ti_3_CN atomic layer modifies its nanotribological behavior compared to Ti_3_C_2_. Using friction force microscopy and peak force quantitative nanomechanics , we find that Ti_3_CN exhibits a notable increase in friction along with higher adhesion and energy dissipation, which we attribute to enhanced hydroxyl termination, stronger surface dipole interactions, and hydrogen bonding. X-ray photoelectron spectroscopy further reveals that nitrogen incorporation leads to greater electron withdrawal from titanium atoms, resulting in a higher oxidation state and altered surface chemical functionality. These results provide mechanistic insight into how X-element chemistry influences the tribological performance of MXenes, highlighting the importance of surface composition in designing 2D materials for specific applications.

## Graphical abstract

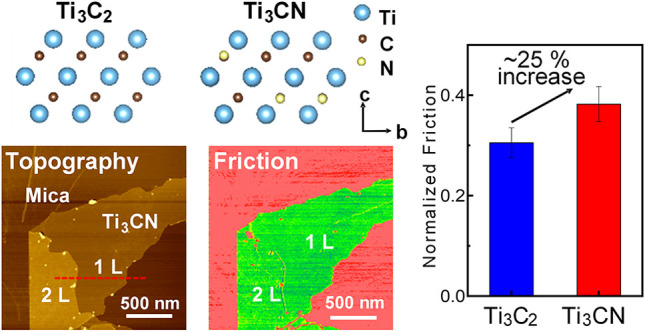

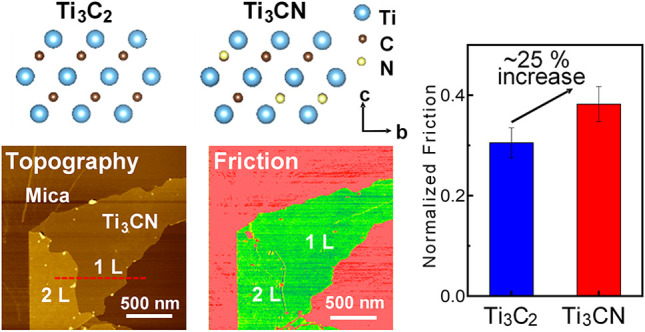

The exfoliation of single-layer graphene from graphite represented a groundbreaking milestone in materials science, igniting extensive research into graphene and other two-dimensional (2D) materials.^1^ Over the subsequent two decades, advancements in methodologies for manipulating 2D materials, including boron nitride and transition-metal chalcogenides, have been achieved through techniques such as exfoliation and chemical deposition.^2,3^ The exceptional properties of these 2D materials have continued to drive significant interest and research in multiple scientific fields.^4–7^

MXenes are a family of 2D transition-metal carbides, nitrides, and carbonitrides that have attracted significant attention since their discovery in 2011.^8^ These materials are typically synthesized by selectively etching out elements from layered ternary carbide or nitride precursors, known as MAX phases,^9^ resulting in structures with the general formula M_*n*+1_X_*n*_T_*x*_. Here, M represents an early transition metal (such as titanium or molybdenum), X is carbon or nitrogen, and T_*x*_ denotes surface terminations, typically consisting of −OH, −O, and −F in the case of wet chemical etching.^10^ MXenes possess excellent electrical conductivity, similar to bulk carbides and nitrides of transition metals, and they exhibit hydrophilicity thanks to their surface terminations. MXenes’ unique combination of properties makes them suitable for various applications, such as energy storage,^11^ water purification,^12^ electromagnetic interference shielding,^13^ sensing,^14^ and catalysis.^15^ Beyond their notable electronic and chemical properties, MXenes exhibit desirable tribological characteristics, including low friction coefficients and high wear resistance, primarily attributed to their layered structure, high mechanical properties, and tunable surface terminations, making them highly promising for tribological applications such as triboelectric nanogenerators.^16–19^

While materials like graphene and MoS_2_ have been extensively studied for their tribological properties at the nanoscale, research on the nanotribological characteristics of MXenes remains in its initial stages. In graphene, hydroxyl (-OH) terminations have been shown to significantly influence friction by modifying the interlayer interactions and adhesion energy. For instance, studies have demonstrated that oxidation in graphene leads to a substantial increase in friction, primarily due to the formation of hydrogen bonds in the interlayer space and the transition from sp^2^ to sp^3^ hybridization.^20^ Density functional theory calculations further predict that atomic-scale friction in graphene oxide is significantly higher than that in pristine graphene, highlighting the dominant role of surface terminations in determining tribological behavior.^21^ Similarly, preliminary friction studies have demonstrated that the friction mechanisms of 2D Ti_3_C_2_T_*x*_ MXenes are governed by surface termination rather than by layer dependence. Annealing MXenes has been shown to reduce OH terminations, significantly lowering friction.^22^ Furthermore, a comparative study on the nanoscale friction and adhesion of Ti_3_C_2_ and Nb_2_C MXenes (in the following, we omit T_*x*_ from the MXene formulas for simplicity, but the reader should understand that all MXenes discussed in this paper have surface terminations) revealed that Nb_2_C exhibits lower friction and adhesion due to differences in the surface dipole moment density. Both MXenes show reduced friction and adhesion with increasing temperature.^23^

Recent reviews have provided comprehensive overviews of MXene tribological applications, including synthesis and lubricating performances,^24^ covalent functionalization strategies,^25^ and the tribological potential and future perspectives of MXenes.^26,27^ While these works summarize significant progress in both fundamental understanding and practical applications, the influence of the X element (e.g., C vs N) on MXenes’ surface chemistry and resulting nanotribological behavior remains largely unexplored. This underscores a critical research gap because the X element defines the structural backbone of MXenes and modulates key surface characteristics, including electronic distribution, oxidation states, and terminal group interactions^28–30^ —all of which impact friction. In this study, we aim to address this gap by systematically comparing Ti_3_C_2_ and Ti_3_CN, focusing on how differences in the X element affect surface dipole interactions, oxidation behavior, and functional group distribution. To the best of our knowledge, this work represents the first comparative investigation that directly links the nature of the X element to the nanoscale frictional behavior through a detailed surface chemical analysis. These insights offer a deeper understanding of the MXene surface chemistry and provide design guidelines for the development of advanced nanolubricants and triboelectric energy harvesters.

2D Ti_3_C_2_ and Ti_3_CN MXenes were synthesized using the mixed acid (HF/HCl) etching method.^31^ More detailed sample preparation is described in the Supporting Information.

Figure 1a shows the schematic diagram of a typical M_3_X_2_ MXene, where X (C or N) atoms are located between three layers of hexagonally close-packed M (Ti) atoms. The X-ray diffraction (XRD) patterns in Figure 1b clearly show the disappearance of characteristic MAX phase peaks in Ti_3_C_2_ (∼39°)^32^ and Ti_3_CN (∼39.5°).^33^ This confirms the successful etching of aluminum layers and subsequent delamination.^34,35^ Notably, XRD patterns of Ti_3_C_2_ MXene include a (211) Si peak at 33.1°, which comes from the substrate (PDF 01-078-6300). Both MXenes exhibit a prominent (002) diffraction peak, appearing at 2θ = 6.55° for Ti_3_C_2_ and at 2θ = 7.38° for Ti_3_CN, respectively. Based on Bragg’s law (λ = 2*d* sin θ), the *d*-spacing values calculated from the (002) plane are 13.5 Å for Ti_3_C_2_ and 11.9 Å for Ti_3_CN. The larger *d*-spacing value observed in Ti_3_C_2_ indicates the insertion of an additional monolayer of water molecules and reduced interlayer interactions relative to Ti_3_CN. The shift of the (002) peaks from 2θ = 9.70° (Ti_3_AlC_2_) to 6.55° (Ti_3_C_2_) and from 2θ = 9.48° (Ti_3_AlCN) to 7.38° (Ti_3_CN) also confirms MXene formation (Figure S1a). Higher-order (00*l*) diffraction peaks are present in Figure S1b, in positions that closely match those reported in the literature.^36^ Parts c and d of Figure 1 show high-resolution transmission electron microscopy (HRTEM) bright-field images of Ti_3_C_2_ and Ti_3_CN, respectively. It can be observed that the Ti atoms on the top layer of Ti_3_C_2_ MXene are in a hexagonal close-packed structure. In contrast, the structure of Ti_3_CN appears to exhibit some distortion, likely due to the presence of both C and N atoms with differing atomic radii. The lattice spacing is measured at 0.23 nm for Ti_3_C_2_ and 0.218 nm for Ti_3_CN, aligning well with previously reported values.^37,38^ Elemental analysis of Ti_3_C_2_ and Ti_3_CN was conducted using energy-dispersive X-ray spectroscopy (EDS) mapping. For Ti_3_C_2_ (Figure S2), Ti, C, O, and F were observed, while for Ti_3_CN (Figure S3), the presence of N was also confirmed. This indicates that, in the case of Ti_3_CN, C and N coexist in the lattice, and both MXenes have O and F terminations.

**Figure 1:**
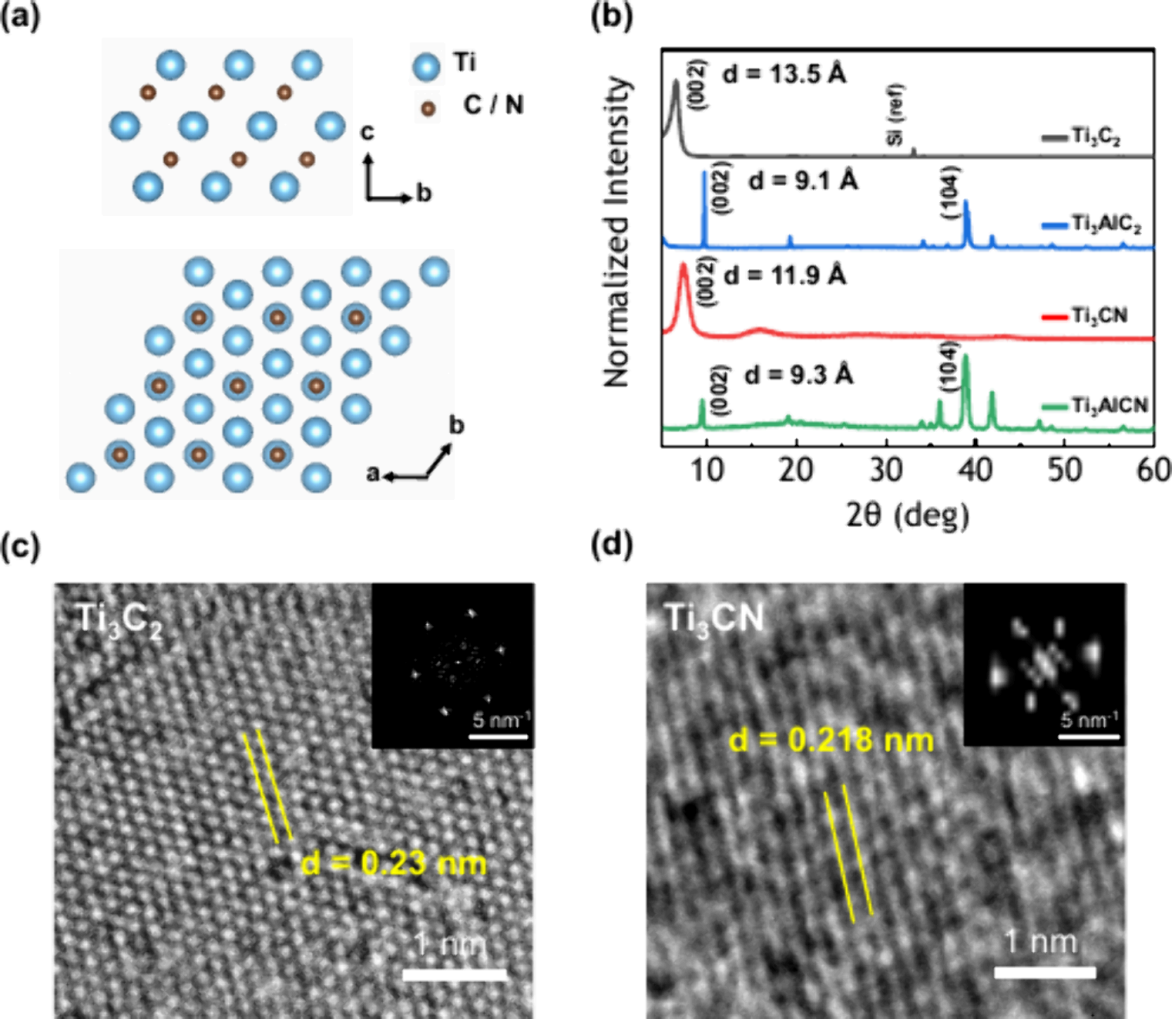
Characterization of Ti_3_C_2_ and Ti_3_CN MXenes. (a) Geometric scheme of a M_3_X_2_ MXene. (b) XRD analysis. HRTEM and FFT (inset) images of (c) Ti_3_C_2_ and (d) Ti_3_CN.

We measured the nanoscale friction force on monolayer flakes of Ti_3_C_2_ and Ti_3_CN within the elastic deformation regime. For friction force microscopy (FFM) measurement, freshly exfoliated mica (Ted Pella, grade V1) with dimensions of 0.5 cm × 0.5 cm was spin-coated with Ti_3_C_2_ and Ti_3_CN MXene, each diluted 200 times with deionized water at 3000 rpm for 30 s. A PPP-LFMR (Nanosensors) cantilever for the contact mode was used, with the spring constant determined to be 0.21 N/m through deflection sensitivity from force–distance curves and thermal tuning. Atomic force microscopy (AFM; Bruker, Multimode 8) in contact mode was used to measure each MXene flake area with a normal force of 10 nN. The friction force was measured as half of the difference in the lateral signal obtained in the trace and retrace directions. The friction was calibrated using a TGF11 silicon calibration grating sample (MikroMasch).^39^

Parts a and c of Figure 2 depict the topography of monolayer Ti_3_C_2_ and Ti_3_CN deposited on mica, and parts b and d of Figure 2 depict friction images corresponding to the topography of Ti_3_C_2_ and Ti_3_CN, respectively. The friction values are obtained from the difference in the lateral signals (Figure S4) between the trace and retrace directions.^40^ To minimize tip effects during measurements, the same cantilever was used for each sample, and the friction was normalized to the unchanged friction of the mica surface observed during measurements.^41,42^ Figure 2e shows the height (above) and friction (below) line profiles for Ti_3_C_2_ (black) and Ti_3_CN (red). By observing the step height between the mica substrate and MXene flakes, we determined monolayer thicknesses of approximately ∼0.8 nm for Ti_3_C_2_ and ∼0.9 nm for Ti_3_CN, with the slightly greater thickness of Ti_3_CN likely being attributed to variations in the quantity and type of surface-terminated species. Despite this difference, the surface roughnesses of both Ti_3_C_2_ (∼70 pm) and Ti_3_CN (∼70 pm) were measured to be very low and comparable to those of typical 2D materials.^43^ Upon examination of the friction line profiles, both types of MXenes exhibit significantly lower friction compared to the mica substrate and show the difference in friction between Ti_3_C_2_ and Ti_3_CN. When normalized to the friction of the mica substrate (set to 1), Ti_3_C_2_ shows a friction value of 0.302 ± 0.03, while Ti_3_CN shows a friction value of 0.382 ± 0.034. This indicates that Ti_3_CN exhibits friction that is approximately 25% higher than that of Ti_3_C_2_ (Figure 2f).

**Figure 2:**
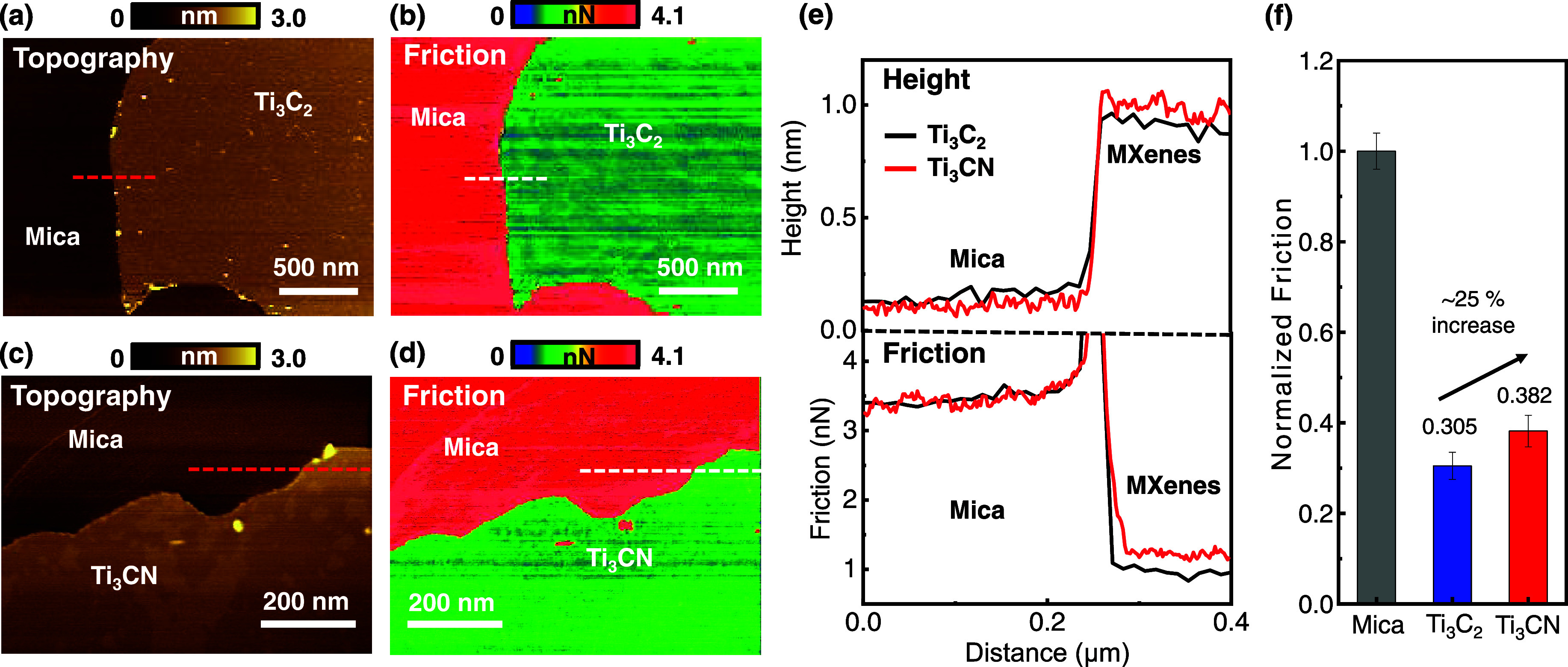
Friction force measurement of Ti_3_C_2_ and Ti_3_CN MXenes. (a) Topography and (b) friction images of Ti_3_C_2_. (c) Topography and (d) friction images of Ti_3_CN. (e) Height (top) and friction (bottom) profiles of Ti_3_C_2_ and Ti_3_CN MXenes. (f) Normalized friction of Ti_3_C_2_ and Ti_3_CN.

We also observed the friction as a function of the number of layers. Parts a and b of Figure 3 show the topography and corresponding friction images of Ti_3_C_2_ with 1–3 layers, and parts d and e of Figure 3 present the topography and friction images of Ti_3_CN with 1 and 2 layers. The thickness of each Ti_3_C_2_ (∼1.1 nm) and Ti_3_CN (∼1.0 nm) layer can be observed through the line profile (Figure S5a,b). The slightly larger thickness of Ti_3_C_2_ compared to Ti_3_CN is in agreement with the lattice spacing results from the XRD data. Although the exact height value differs from the *d* spacing due to uncertainties in the AFM height measurements,^44^ the trend remains consistent.

**Figure 3:**
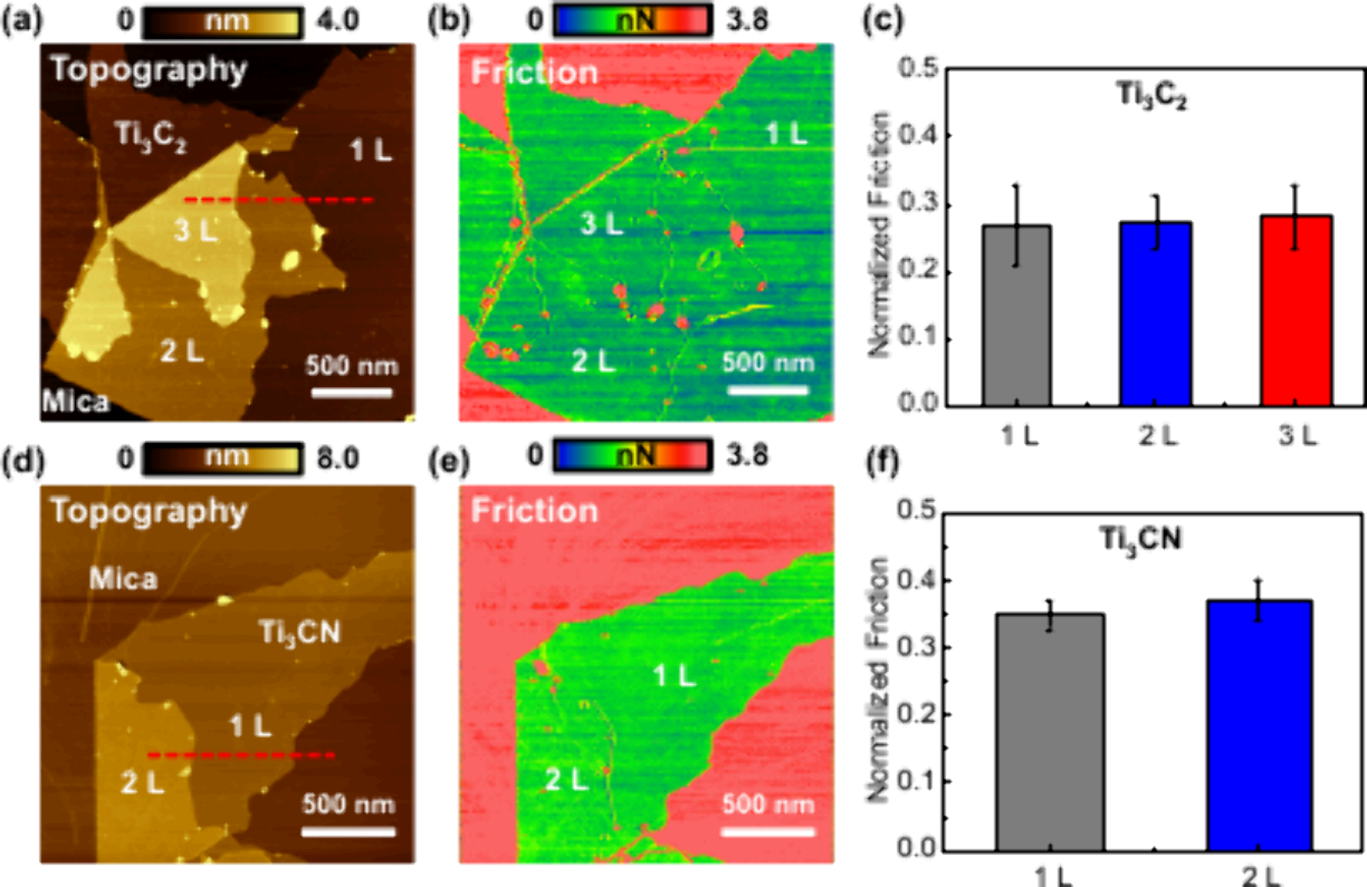
Layer-independent friction of Ti_3_C_2_ and Ti_3_CN MXenes. (a) Topography and (b) friction of Ti_3_C_2_. (c) Normalized friction of monolayer, bilayer, and trilayer Ti_3_C_2_ MXenes. (d) Topography and (e) friction of Ti_3_CN. (f) Normalized friction of monolayer and bilayer Ti_3_CN MXenes.

Regarding friction, Ti_3_CN consistently exhibits higher friction than Ti_3_C_2_ across all tested layer numbers. Interestingly, both MXenes show minimal variation in friction with the increasing number of layers (Figure 3c,f), indicating layer-independent tribological behavior. This phenomenon has previously been reported for Ti_3_C_2_-based MXenes, where strong interlayer coupling, reinforced by surface terminations, was identified as the primary mechanism behind thickness-independent friction.^22^ Our findings extend this understanding by demonstrating that Ti_3_CN also exhibits such layer-independent frictional behavior, suggesting that robust interlayer interactions may be an intrinsic feature of MXenes, regardless of the specific X element.

To investigate other nanotribological properties, such as adhesion and energy dissipation, we used silicon wafers as the reference material for peak force measurements. Considering the 10 nN contact force used in FFM, we also set the peak force to 10 nN in peak force quantitative nanomechanical mapping (PF-QNM).^45^ Ti_3_C_2_ and Ti_3_CN MXenes, each diluted 200 times with deionized water, were prepared by spin-coating onto Si (100) wafers at 1500 rpm for 60 s. The samples were then subjected to overnight vacuum drying to remove the residual moisture. AFM (Bruker, Multimode 8) in peak force tapping mode was utilized using a PPP-EFM cantilever (Nanosensors). The spring constant was determined to be 2.58 N/m, derived from a deflection sensitivity of 48.48 nm/V obtained from force–distance curves on the Si substrate and a resonance frequency of 70.89 kHz obtained from thermal tuning. Based on this, a constant peak force of 10 nN was applied to measure adhesion and energy dissipation. Silicon was chosen as the substrate due to its higher stiffness (∼180 GPa) and sufficient surface smoothness, making it easier to measure the intrinsic mechanical properties of the MXene samples rather than characteristics influenced by the substrate, as would be more likely with a lower-modulus material like mica.

Parts a–f of Figure 4 show the topography, adhesion, and energy dissipation maps of Ti_3_C_2_ and Ti_3_CN. Parts b and e of Figure 4 show the adhesion of Ti_3_C_2_ (8.25 nN) and Ti_3_CN (8.61 nN), respectively, indicating that Ti_3_CN generally has greater adhesion than Ti_3_C_2_. The observed differences in adhesion between the two materials may be attributed to variations in surface termination, which will be further discussed in subsequent sections through X-ray photoelectron spectroscopy (XPS) analysis. Previous theoretical studies, such as those by Righi et al.,^46^ have shown that MXene adhesion is influenced by the nature of the surface terminations and the interlayer interactions. These studies highlighted the significance of surface functional groups in controlling adhesion and lubrication properties, and our findings align with this by showing that Ti_3_CN exhibits stronger adhesion compared with Ti_3_C_2_, likely due to differences in their surface chemistry. Similarly, Parts c and f of Figure 4 display the energy dissipation of Ti_3_C_2_ (367.5 eV) and Ti_3_CN (383.5 eV), showing that Ti_3_CN also has higher energy dissipation compared to Ti_3_C_2_. Parts h and i of Figure 4 present the normalized adhesion and energy dissipation for each material by the silicon substrate, respectively. The data show a trend of increasing adhesion and energy dissipation from silicon to Ti_3_C_2_ and to Ti_3_CN.

**Figure 4:**
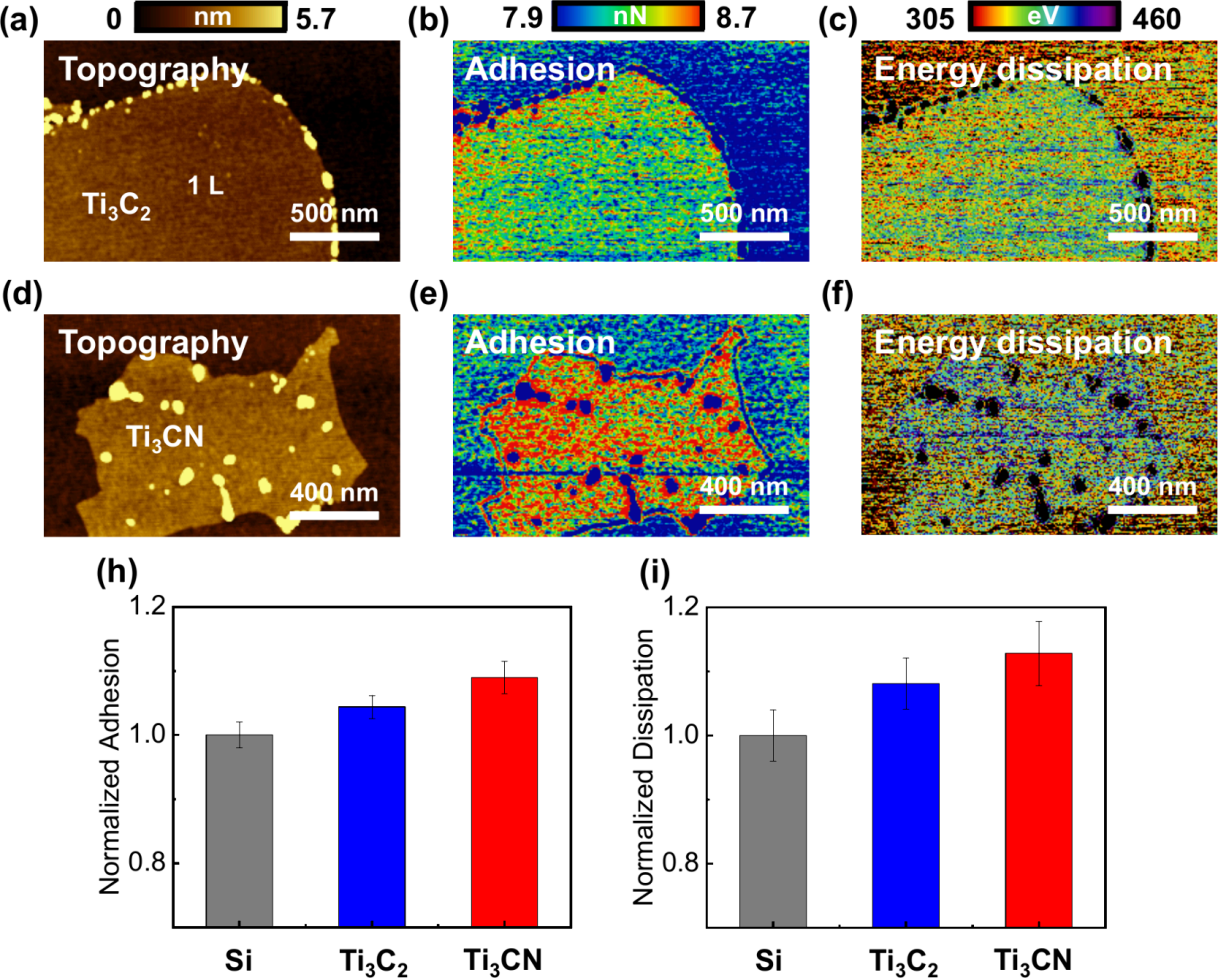
Adhesion and energy dissipation measurement of Ti_3_C_2_ and Ti_3_CN MXenes by using peak-force-mode AFM. (a) Topography, (b) adhesion, and (c) energy dissipation maps of Ti_3_C_2_. (d) Topography, (e) adhesion, and (f) energy dissipation maps of Ti_3_CN. (h) Normalized (by comparison to Si) adhesion of Si, Ti_3_C_2_, and Ti_3_CN. (i) Energy dissipation of Si, Ti_3_C_2_, and Ti_3_CN normalized by Si.

We conducted XPS measurements to identify the surface chemical properties of Ti_3_C_2_ and Ti_3_CN and their relationship with the nanotribological properties. Figure S6a displays the XPS survey spectra of Ti_3_C_2_ and Ti_3_CN, respectively. In the XPS survey spectra, the disappearance of the specific Al peak from the MAX phase indicates successful etching and delamination. While the nitrogen 1s peak around 396.9 eV is negligible in Ti_3_C_2_, a distinct nitrogen 1s peak is observed in Ti_3_CN. Compared to Ti_3_C_2_, Ti_3_CN also shows a decrease in the intensity of the F 1s peak and a relative increase in the intensity of the O 1s peak. This suggests a decrease in the proportion of fluorine among the surface terminations and an increase in oxygen species. Figure S6b presents the C 1s spectra for Ti_3_C_2_ and Ti_3_CN. The binding energies of Ti_3_C_2_ and Ti_3_CN were calibrated using the C–C bond peak at 284.8 eV. The peak observed at a lower binding energy of 282.2 eV, attributed to the C–Ti bond, is sharp in Ti_3_C_2_, whereas it appears broader in Ti_3_CN. This broadening suggests that, with the addition of nitrogen, the Ti atoms bound to carbon exhibit a greater diversity of oxidation states in Ti_3_CN. In Figure S6c, the Ti 2p spectra of both Ti_3_C_2_ and Ti_3_CN were deconvoluted to satisfy the 2:1 area ratio of Ti 2p_3/2_ and Ti 2p_1/2_ spin–orbit splitting, considering the three states (Ti^2+^, Ti^3+^, and Ti^4+^) within the 450–470 eV binding energy range. For Ti_3_C_2_, the 2p_3/2_ spectra at 454.9, 456.1, and 459.3 eV were assigned to Ti^2+^, Ti^3+^, and Ti^4+^, respectively. For Ti_3_CN, the 2p_3/2_ spectra at 455.4, 456.8, and 459.4 eV were assigned to Ti^2+^, Ti^3+^, and Ti^4+^, respectively. Ti_3_CN shows a slight increase in the binding energy of the Ti 2p peak associated with the lattice, which is attributed to the higher electronegativity of nitrogen.

To further validate the overall shift toward higher binding energies in the Ti 2p spectra, Kelvin probe force microscopy (KPFM) measurement was performed using an atomic force microscope (Keysight 5500). A conductive, noncontact cantilever (PPP-EFM, Nanosensors) coated with Pt/Ir was used as the probe, operating at a resonance frequency of 72.5 kHz. To calibrate the tip’s work function, contact potential difference (CPD) measurements were conducted on a polycrystalline gold reference material before and after each sample analysis.

The topography and surface potential images of Ti_3_C_2_ (Figure 5a) and Ti_3_CN (Figure 5b) were obtained. The *V*_CPD_ value is calculated as follows: $V_{\mathrm{CPD}}=\frac{\Phi_{\mathrm{tip}}-\Phi_{\mathrm{sample}}}{e}$ . Here, Φ_tip_ and Φ_sample_ denote the respective work function values of the tip and sample, while *e* represents the charge of an electron.^47^ As shown in Figure 5c, Ti_3_CN has a higher CPD than Ti_3_C_2_, corresponding to a lower work function for Ti_3_CN. This observation supports the conclusion that nitrogen induces stronger electron withdrawal from titanium, resulting in a higher oxidation state and increased binding energies in the Ti 2p spectra. Additionally, a noticeable increase in the peak intensity around 459.8 eV is observed in Ti_3_CN, which is attributed to titanium atoms in a more oxidized state due to the influence of oxygen-containing terminated species. This increase in the titanium oxidation state is further confirmed by the analysis of oxygen species binding to the surface titanium. The O 1s spectra of Ti_3_C_2_ and Ti_3_CN were deconvoluted to identify the different oxygen species present on the surface, providing insight into the role of oxygen in the oxidation process. In Figure 5d,e, the O 1s spectra of Ti_3_C_2_ and Ti_3_CN were deconvoluted into three states: Ti–O (bridge), Ti–O (FCC), and Ti–O–H at 530.0 530.9, and 532.1 eV in both Ti_3_C_2_ and Ti_3_CN. Based on the peak areas of these assignments and the O 1s spectra, we analyzed the stoichiometric ratios of the terminated oxygen species on the surface titanium of each MXene. Figure 5f shows the stoichiometric ratios of the terminated species for Ti_3_C_2_ and Ti_3_CN. Compared to Ti_3_C_2_, Ti_3_CN exhibited a decrease in Ti–O (bridge) and an increase in Ti–O bindings at the FCC site and Ti–OH bindings. In the case of Ti_3_CN, Ti has a more oxidized state. This results in three surface chemical differences. First, both oxygen and fluorine, as terminating species, achieve the most stable state at the FCC site on the Ti surface, leading to competition for this site. In this context, Ti in Ti_3_CN, which has a higher oxidation state due to the presence of nitrogen, is likely to prefer oxygen over the more electronegative fluorine. Furthermore, unlike fluorine, which binds exclusively at the FCC site, oxygen can bind at both the FCC site and the slightly unstable bridge site.^48^ In Ti_3_CN, as oxygen’s competitiveness at the FCC site increases, Ti–O (FCC) binding predominantly forms, resulting in a decreased tendency for Ti–O (bridge) binding. Finally, by comparing the electron-withdrawing strength of the terminating species −F, −O, and −OH, it is observed that the amount of OH termination, which has a relatively weaker electron-withdrawing tendency, increases. Through the comparison of Ti_3_C_2_ and Ti_3_CN, we examined how the X elements of MXenes affect their surface chemical properties. Additionally, we found that the increase in the total amount of terminated species and the increase in oxygen binding at the FCC sites on the Ti_3_CN surface, compared to those of Ti_3_C_2_, clearly contribute to a larger surface dipole moment. The increased hydroxyl termination also leads to stronger interactions with the AFM tip, such as hydrogen bonding, resulting in increased surface adhesion and energy dissipation. According to previous studies, the type of terminated species bonded to titanium affects the minimum energy pathway (MEP). Hydroxyl termination has a higher MEP than fluorine or oxygen, significantly impacting the frictional properties of the MXene surface. Our results show that the presence of nitrogen leads to a preference for hydroxyl termination at the Ti bridge site in Ti_3_CN compared to Ti_3_C_2_. This can be attributed to an approximately 25% increase in the friction observed in Ti_3_CN.

**Figure 5:**
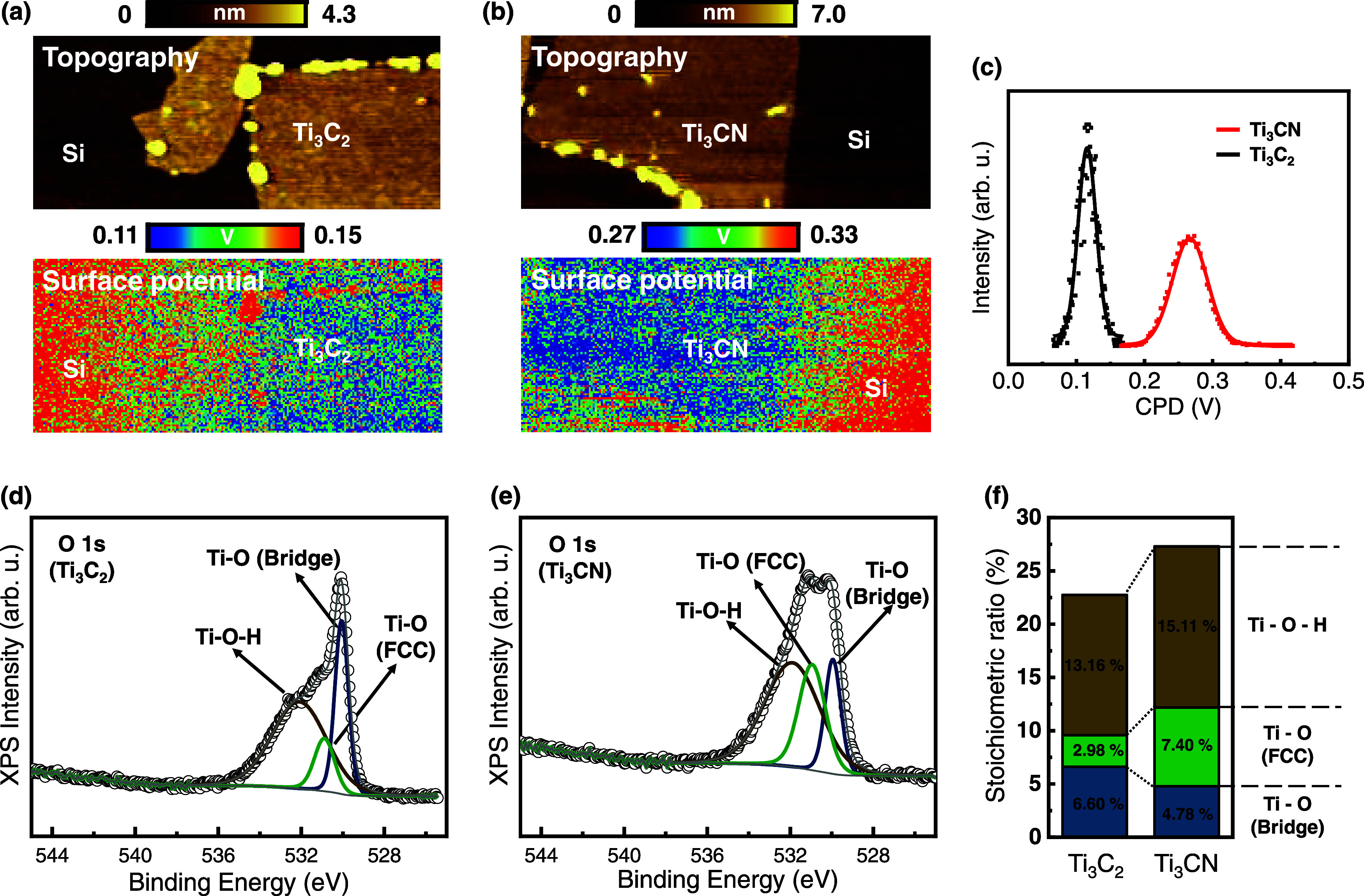
KPFM measurement and XPS spectra of Ti_3_C_2_ and Ti_3_CN MXenes. Topography and surface potential images of (a) Ti_3_C_2_ and (b) Ti_3_CN. (c) CPD of Ti_3_C_2_ and Ti_3_CN. O 1s of (d) Ti_3_C_2_ and (e) Ti_3_CN. (f) Stoichiometric ratio of oxygen termination species.

In summary, this study demonstrates that the X sublattice elements in MXenes significantly influence their surface chemistry and nanotribological properties. XPS analysis revealed that the Ti_3_CN sample used in this study exhibits more hydroxyl terminations and an increased oxidation state of Ti atoms compared to Ti_3_C_2_. Our FFM measurements show that Ti_3_CN exhibits ∼25% higher friction than Ti_3_C_2_, a direct consequence of increased hydroxyl (−OH) termination density and stronger surface dipole moments. Prior studies have shown that the friction of Ti_3_C_2_T_*x*_ MXenes decreases after annealing due to the partial removal of −OH surface terminations. Similarly, in Ti_3_CN, the prevalence of hydroxyl groups increases interfacial energy barriers, increasing the MEP for atomic-scale sliding. Additionally, Ti_3_CN shows greater adhesion and energy dissipation than that of Ti_3_C_2_. The increased adhesion in Ti_3_CN is primarily due to its higher surface dipole moment, which arises from the greater number of terminated species on the surface. This increased adhesion contributes to a higher energy dissipation, as observed from the larger area under the force–displacement curve. In conclusion, the surface chemistry differences induced by the X sublattice elements in MXenes play a critical role in determining their nanotribological behavior, with Ti_3_CN exhibiting higher friction, adhesion, and energy dissipation due to its increased hydroxyl terminations and surface dipole moment.

## Supplementary Material



## Notes

Synthesis of MAX phases and MXenes, characterization of Ti_3_C_2_ and Ti_3_CN MXenes (XRD, XPS, and C_s_-STEM), EDS mapping of Ti_3_C_2_ and Ti_3_CN MXenes, height line profiles of layered Ti_3_C_2_ and Ti_3_CN MXenes, lateral signal, and XPS spectra of Ti_3_C_2_ and Ti_3_CN (PDF)

## Abbreviations

CPD: contact potential difference
C_s_-STEM: spherical aberrations corrected scanning TEM
EDS: energy-dispersive X-ray spectroscopy
FFM: friction force microscopy
KPFM: Kelvin probe force microscopy
MEP: minimum energy pathway
TEM: transmission electron microscopy
2D: two-dimensional
XPS: X-ray photoelectron spectroscopy
XRD: X-ray diffraction
