# Supplementary material for: Influence of X Elements on the Tribological Properties and Surface Chemistry of MXene Atomic Layers
Source: Nano Lett. 2025 Jun 7;25(24):9817–24. doi: 10.1021/acs.nanolett.5c02270 (PMC12239071; doi:10.1021/acs.nanolett.5c02270)
Supplement: Supplementary file 1 [file nl5c02270_si_001.pdf]

Supporting Information

Influence of X-elements on Tribological Properties and Surface Chemistry of MXene Atomic Layers

Hong Yeon Yoon[1+], Hyunjoon Yoo[2+], Manmatha Mahato[2], Jong Hun Kim[3], Sokhna Dieng[4], Chi Won Ahn[5], Yury Gogotsi[4], Il-Kwon Oh[2*], and Jeong Young Park[1*]*

[1]Department of Chemistry, Korea Advanced Institute of Science and Technology (KAIST), Daejeon, 34141, Republic of Korea

[2] National Creative Research Initiative for Functionally Antagonistic Nano-Engineering, Department of Mechanical Engineering, Korea Advanced Institute of Science and Technology (KAIST), Daejeon 34141, Republic of Korea

[3]Department of Physics, Inha University, Incheon, 22212 Republic of Korea

[4]Department of Materials Science & Engineering, and A.J. Drexel Nanomaterials Institute, Drexel University, Philadelphia, Pennsylvania 19104, United States

[5]National Nanofab Center (NNFC), Korea Advanced Institute of Science and Technology (KAIST), 291 Daehak-ro, Yuseong-gu, Daejeon 34141, Republic of Korea

Synthesis of MAX Phases and MXenes

The synthesis of $Ti_3C_2T_x$ and Ti_3CNT_x MXenes was carried out through the etching and delamination of their respective MAX phases, Ti_3AlC_2 and Ti_3AlCN.[1-3] The process began with the preparation of the MAX phases.

For the synthesis of Ti_3AlC_2, precursor powders of titanium carbide (TiC), titanium (Ti), and aluminum (Al) were mixed in a stoichiometric ratio of 2:2.2:1.25. This mixture underwent ball milling in high-density polyethylene containers using 3 mm yttrium-stabilized zirconium balls, maintaining a powder-to-ball weight ratio of 1:2. The milling process was carried out at 70 rpm for 15 hours. After milling, the powder blend was transferred to alumina crucibles and sintered at 1380°C for 2 hours under an argon atmosphere, with both heating and cooling rates set at 3°C/min. The resulting ceramic block was initially milled using a titanium carbide bit and further ground using a porcelain mortar and pestle. The powder was then sieved through a 400-mesh sieve to achieve a particle size below 38 µm. To remove intermetallic impurities and unreacted elements, the MAX phase was subjected to acid treatment with 9 M hydrochloric acid for 24 hours. The acid-treated powder was recovered by vacuum filtration through a 5 µm polycarbonate membrane and thoroughly washed with deionized (DI) water until the pH exceeded 6. Finally, the purified MAX phase powder was dried overnight in a vacuum oven at 60°C.

The synthesis of Ti_3AlCN followed a similar procedure. Precursor powders of titanium (Ti), aluminum nitride (AlN), and graphite were mixed in a stoichiometric ratio of 3:1:1. The mixture was subjected to ball milling under the same conditions as Ti_3AlC_2. After milling, the powder was sintered at 1500°C for 2 hours under an argon atmosphere, maintaining a heating and cooling rate of 3°C/min. The post-processing steps, including grinding, sieving, acid treatment, filtration, washing, and drying, were identical to those used for Ti_3AlC_2.

Following the synthesis of the MAX phases, etching was performed to selectively remove the aluminum layers and obtain the corresponding MXene structures. For $Ti_3C_2T_x$, an etchant solution consisting of 12 mL of hydrochloric acid (HCl, 36%), 2 mL of hydrofluoric acid (HF, 49%), and 6 mL of DI water was prepared in a high-density polyethylene bottle. To this solution, 1 g of Ti_3AlC_2 MAX phase powder was gradually added under gentle stirring. The mixture was stirred at 340 rpm and maintained at 35°C for 24 hours to facilitate etching. After the etching process was complete, the reaction mixture was centrifuged at 3500 rpm for 5 minutes, and the supernatant was discarded. The sediment was washed and resuspended in DI water, with this step repeated several times until reaching a neutral pH.

For Ti_3CNT_x, a similar etching process was followed, using the same etchant solution and reaction conditions. After etching, the MAX phase residues were centrifuged and washed following the same procedure as for $Ti_3C_2T_x$ to eliminate any remaining reaction byproducts.

The delamination process was then performed to separate individual MXene nanosheets. For $Ti_3C_2T_x$, the etched sediment was resuspended in 20 mL of DI water containing 1 g of lithium chloride (LiCl). The dispersion was stirred at 340 rpm and maintained at 35°C for 12 hours. For Ti_3CNT_x, delamination was carried out using the same delaminant solution and reaction conditions. After delamination, both dispersions were centrifuged at 3500 rpm for 5 minutes, and the process was repeated several times to isolate the delaminated MXene nanosheets.

Characterization of Ti_3C_2 and Ti_3CN MXenes (XRD, XPS, Cs-STEM)

Unless otherwise specified, all physicochemical analyses were performed on MXene samples spin-coated onto Si (100) wafers. The MXene samples, diluted 100 times with deionized water after cleaning, were spin-coated at 1000 rpm for 60 seconds. To remove any remaining water, the samples were dried in a vacuum oven for 12 hours.

XRD patterns were obtained using a RIGAKU Smart Lab High-Resolution Powder X-Ray Diffractometer with Cu Kα1 radiation. For each sample, the step speed was 3°/min, the step size was 0.01°, and the power settings were 45 kV and 200 mA. Surface chemical analysis was performed using XPS with a Thermo Scientific NEXSA-G2 instrument. The X-ray source used for XPS analysis was Al K_α with a beam size of 400 μm. Atomic-scale imaging was conducted using a JEOL JEM-ARM200F Cs-corrected scanning-transmission electron microscope (Cs-STEM) operated at an acceleration voltage of 200 kV. For single-layer flake analysis, a dilute MXene dispersion was sonicated, drop-coated onto a TEM grid, and dried under a vacuum oven for 6 hours. Chemical composition mapping and analysis were performed using the EDS integrated with the Cs-STEM instrument.

References

1. Downes, M., Shuck, C.E., McBride, B., Busa, J., Gogotsi, Y., 2024. Comprehensive synthesis of $Ti_3C_2T_x$ from MAX phase to MXene. *Nature Protocols* **2024**, 19. 1807–1834

2. Shuck, C.E., Ventura-Martinez, K., Goad, A., Uzun, S., Shekhirev, M., Gogotsi, Y., Safe Synthesis of MAX and MXene: Guidelines to Reduce Risk During Synthesis. *ACS Chem. Health Saf.* **2021,** 28, 326−338

3. Zhang, T., Shuck, C.E., Shevchuk, K., Anayee, M., Gogotsi, Y., 2023. Synthesis of Three Families of Titanium Carbonitride MXenes. *J. Am. Chem. Soc.* **2023,** 145, 22374−22383

Supplementary Figures

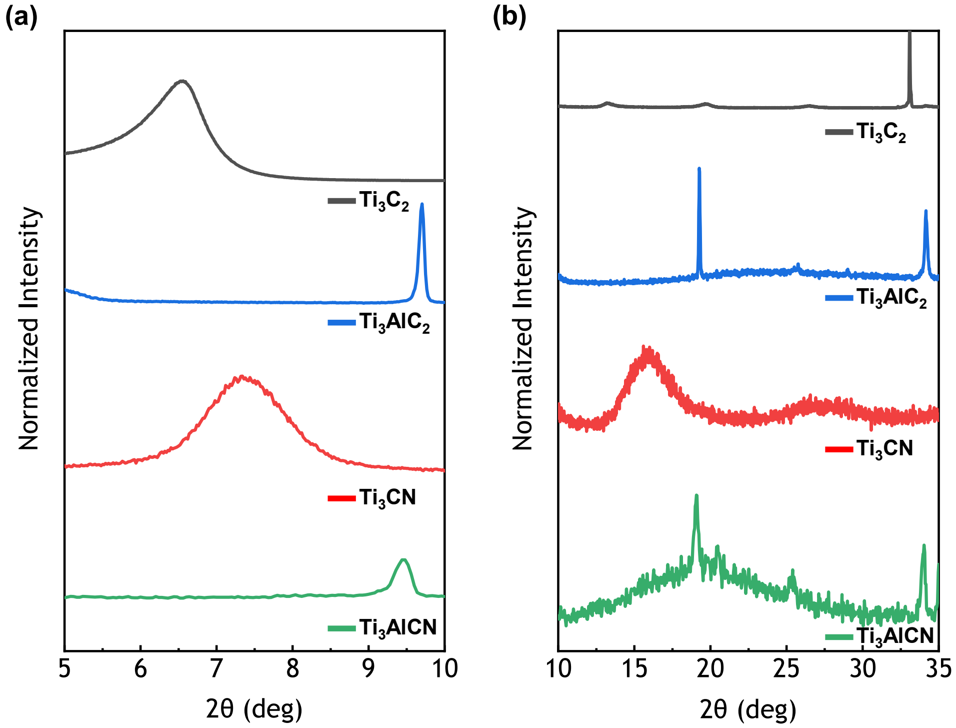

Figure S1. XRD profiles of MAX phases and MXenes: (a) Shifts in the (002) diffraction peaks after etching and delamination processes and (b) higher-order (00l) diffraction peaks demonstrating structural characteristics.

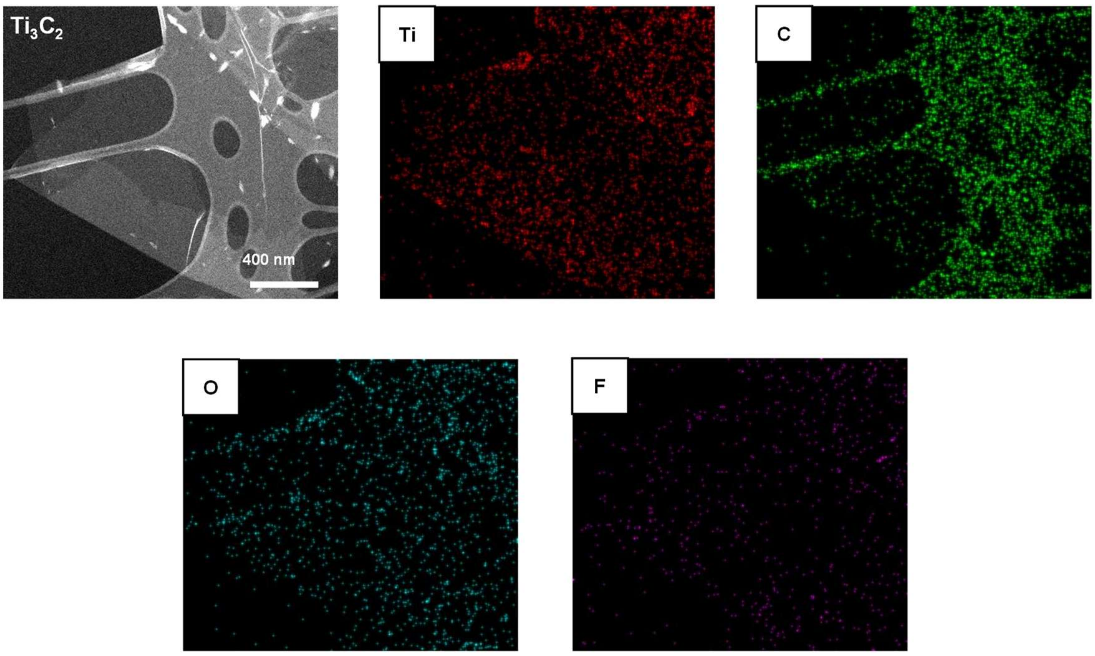

Figure S2. EDS maps of Ti_3C_2.

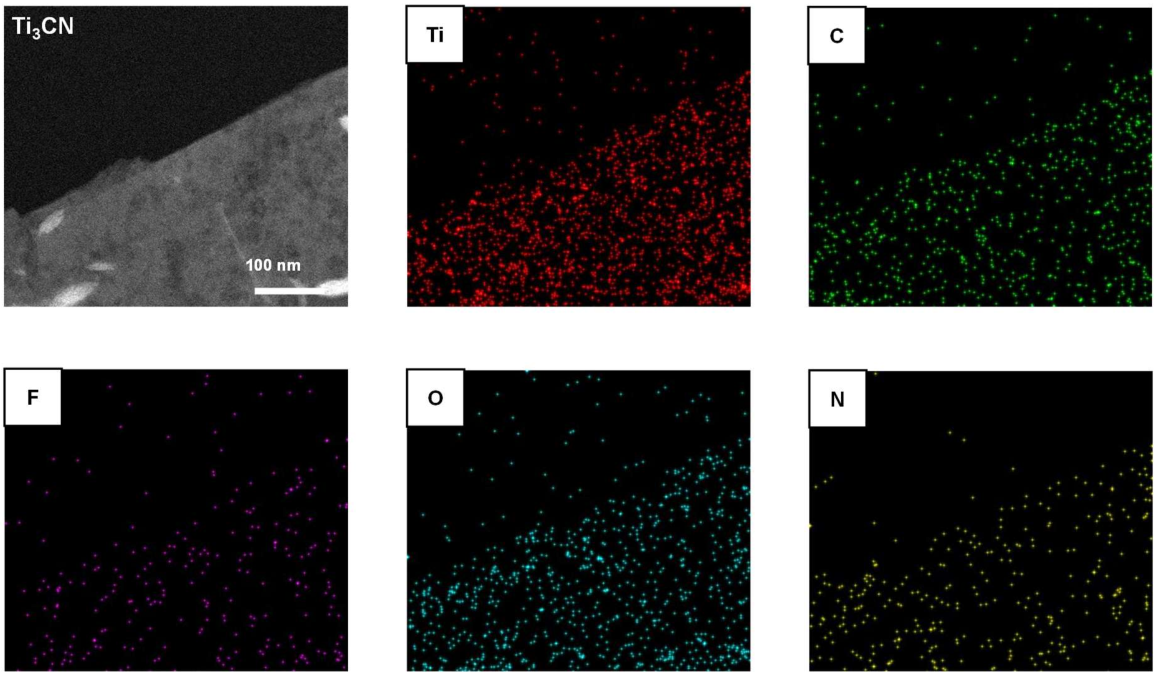

Figure S3. EDS maps of Ti_3CN.

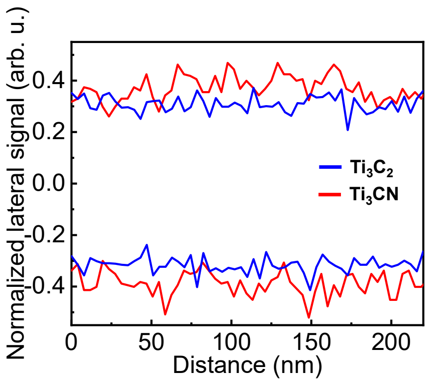

Figure S4. Lateral signal of Ti_3C_2 and Ti_3CN

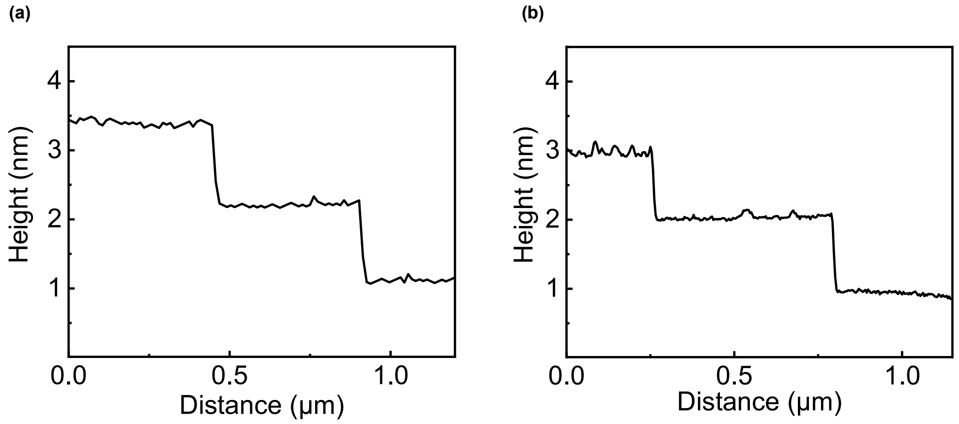

Figure S5. Height profile of (a) Ti_3C_2 and (b) Ti_3CN

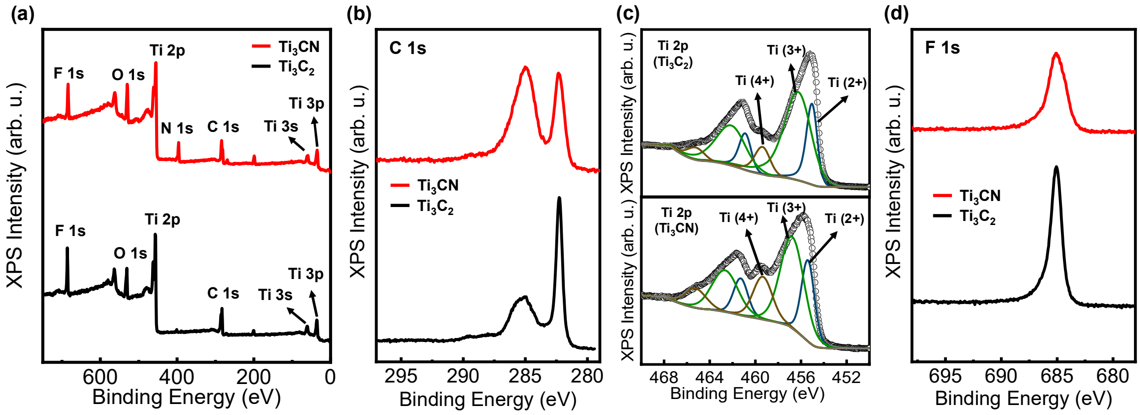

Figure S6. XPS spectra of Ti_3C_2 and Ti_3CN. (a) XPS survey spectra. (b) C 1s spectra. (c) Ti 2p spectra. (d) F 1s spectra.
